# The impact of drought stress under different soil matrices on physiological characteristics of soybean seedlings

**DOI:** 10.1093/aobpla/plaf026

**Published:** 2025-07-16

**Authors:** Wei Zhao, Xiyue Wang, Xinhe Wei, Xiaomei Li, Jixuan Sun, Shoukun Dong

**Affiliations:** College of Agriculture, Northeast Agricultural University, No. 600 Changjiang Road, Xiangfang District, Harbin, 150030, China; College of Agriculture, Northeast Agricultural University, No. 600 Changjiang Road, Xiangfang District, Harbin, 150030, China; College of Agriculture, Northeast Agricultural University, No. 600 Changjiang Road, Xiangfang District, Harbin, 150030, China; College of Agriculture, Heilongjiang Agricultural Engineering Vocational College, No. 2 Qunying Street, Limin Economic and Technological Development Zone, Harbin, 150025, China; College of Agriculture, Northeast Agricultural University, No. 600 Changjiang Road, Xiangfang District, Harbin, 150030, China; College of Agriculture, Northeast Agricultural University, No. 600 Changjiang Road, Xiangfang District, Harbin, 150030, China

**Keywords:** legumes, water stress, physiological response, loamy sand, sandy loam

## Abstract

Drought stress can affect the growth of soybean seedlings because soybeans require a large amount of water for growth and development. However, the storage and redistribution of water in the soil are related to the soil’s texture. This experiment used the soybean varieties hefeng46 and heinong84, and studied the effects of four moisture conditions on the content of membrane lipid peroxides, the activities of enzymes and non-enzymatic antioxidants the content, and also the key enzymes of carbon and nitrogen metabolism in soybean seedlings under loamy sand and sandy loam soil conditions. The results suggested that as the duration of drought increased, in loamy sand, under serious drought (SD), the contents of malondialdehyde and proline, as well as the activities of superoxide dismutase and glutamine synthetase, in hefeng46 and heinong84 were significantly increased by 160% and 146%, 1431% and 1924%, 167% and 282%, and 64% and 69%, respectively, compared to the normal water (CK). However, in sandy loam, the hydrolytic direction activity of sucrose synthase in intermediate drought treated hefeng46 and heinong84 was significantly increased by 1247% and 169% compared to the CK, and the content of reduced glutathione was dramatically raised. In contrast, the synthetic direction activity of sucrose synthase in SD treated hefeng46 and heinong84 was significantly decreased by 69% and 70% compared to the CK. The combined results indicated that under drought stress, soybean in sandy loam soil exhibited stronger drought resistance.

## Teaser

In this paper, the effects of four different degrees of drought stress on the physiological metabolism of hefeng46 and heinong84 soybean leaves at seedling stage under two soil substrates of loamy sand and sandy loam were studied. The degree of membrane lipid peroxidation, antioxidant system, carbon and nitrogen metabolism were used to explore the drought resistance of soybean in two soil substrates. The results showed that soybean showed stronger drought resistance in sandy loam.

## Introduction

Soybean (*Glycine max* (L.) Merr.), as a crop rich in unsaturated fatty acids, high-quality protein, and dietary fibre, is widely planted around the world (Mayakrishnan *et al*. 2018). Soybean is relatively sensitive to water at various developmental stages and requires a large amount of water to maintain normal growth and development. The impact of drought stress on growth changes at diverse developmental stages; drought stress during the seedling stage can cause irreversible effects on growth, while water shortage during the flowering and podding stage can lead to reduced yields or even complete crop failure (Lin *et al*. 2020). Water conditions can affect the physiological and biochemical processes of soybean growth and metabolism such as growth metabolism in soybean, reduces photosynthetic capacity, and simultaneously accumulates a large amount of reactive oxygen species (ROS), causing membrane lipid peroxidation and deesterification (Wang *et al*. 2024). Under drought stress, the plant itself will carry out antioxidant regulation, osmotic regulation, and cell membrane structure regulation through a range of physiological variations to reduce damage (Gupta *et al*. 2020; Romano 2021).

Malondialdehyde (MDA) is one of the outcomes of membrane lipid peroxidation and has cytotoxic effects, causing cross-linking of large molecules such as proteins, which can reflect the degree of lipid peroxidation of plant cell membranes. When MDA accumulates in large quantities, it exacerbates damage to the cell membrane, thereby affecting the normal growth of plants (Begum *et al*. 2023). To ensure normal plant growth, the plant body has developed two efficient antioxidant mechanisms: enzymatic and non-enzymatic antioxidants (Rajput *et al*. 2021; Rudenko *et al*. 2023). Superoxide dismutase (SOD) is an antioxidant enzyme in plant cells and the first line of defense against ROS in plants. It can clear free radicals within the cell and protect the body from oxidative damage. On the other hand, reduced glutathione (GSH), as a non-enzymatic antioxidant, is one of the most important metabolites in plant defense systems, utilizing the ascorbic acid-glutathione (ASA-GSH) cycle to clear ROS, playing a significant role in defending against lipid peroxidation caused by free radicals (Jiang *et al.* 2022). Proline (Pro) is not only a significant osmotic regulator in the plant body but also a non-enzymatic antioxidant. It accumulates in large quantities in the plant body during stress and is used to maintain the whole cell membrane and reduce water loss (Faisal and Muhammad 2023).

Plants regulate osmotic pressure by synthesizing and accumulating compatible solutes within the cell, and most compatible solutes are compounds of carbon and nitrogen metabolism (Zulfiqar *et al.* 2020). Research has found that drought can increase the activity of various carbon metabolism enzymes in plants (Du *et al*. 2020b). Research has found that when plants are subjected to stress that blocks glycolysis and the tricarboxylic acid cycle, they activate the pentose phosphate pathway to replace normal aerobic respiration in order to maintain normal growth and development(Yu *et al*. 2023). And 6-phosphogluconate dehydrogenase (6PGDH) is the key rate-limiting enzyme of the pentose phosphate pathway (PPP) (Muñoz-Vargas *et al*. 2020). Sucrose is one of the products of plant photosynthesis and one of the main forms of carbohydrates accumulated and stored. Sucrose synthase catalyses the synthesis and hydrolysis of sucrose (Stein and Granot 2019). Therefore, the activity of 6PGDH and SuSy can reflect the influence of drought on the carbon metabolism of plants.

Research has shown that drought stress reduces the activity of key enzymes in plant nitrogen metabolism, hindering the accumulation and transport of nitrogen (Cao *et al*. 2022). Nitrate reductase (NR) is an enzyme that reduces nitrate to nitrite while releasing energy, providing nitrogen for plants. It is an important rate-limiting and regulatory enzyme in plant nitrogen metabolism. Its activity affects the total nitrogen level of plants. Glutamine synthetase (GS) is another indispensable enzyme in the absorption of nitrogenous substances in plants. NR and GS play a regulatory role in the absorption of nitrogenous substances by plants (Liao *et al*. 2019). Therefore, changes in the activity of NR and GS can reflect the influence of drought on the nitrogen metabolism of plants (Du *et al*. 2020a).

Plant growth is not only affected by elements such as light, temperature, moisture, and accumulated temperature, but also by soil type. Soil is a system with fractal properties, and different types of soil have different textures. The storage and redistribution of water in soil are related to soil texture (Radcliffe *et al*. 2002). Loamy sand and sandy loam are common soil types in China (Xiu *et al*. 2021). Research has shown that factors such as soil hardness, porosity, moisture content, and the size of aggregates have some impact on plant growth (Huang *et al.* 2021). Therefore, exploring the impact of soil type on the physiological characteristics of soybean seedlings under four water conditions and selecting soil types that are more suitable for soybean cultivation have significant practical importance for soybean production.

This test studied the growth conditions of two soybean varieties (hefeng46 and heinong84) in two different soil types (sandy loam and loamy sand), exploring the changes in physiological indicators of soybeans under four water conditions. This research provides theoretical evidence for selecting suitable soil for soybean cultivation.

## Experimental materials and methods

The test took place in a glasshouse at the experimental station within Northeast Agricultural University. The experimental soil consisted of sandy loam and loamy sand (Table 1), and the soybean varieties were hefeng46 and heinong84, both of which are widely planted in Heilongjiang Province, China.

**Table 1.** The information on loamy sand and sandy loam.

Soil matrix	Loamy sand	Sandy loam
Volumetric weight (g/cm^3^)	1.41	1.43
Total porosity (%)	44.1	44.7
Organic content (g/kg)	7.421	7.122
Total nitrogen content (g/kg)	0.59	0.42
Total phosphorus content (g/kg)	0.33	0.28
Total potassium content (g/kg)	28.87	25.86

### Experimental design

In a plastic barrel with a height of 35 cm, a diameter of 30 cm, and a bottom hole, 14 kg of dry soil without weeds and rocks was filled. Six complete and uniform seeds were selected for planting in each barrel. The experiment included two varieties, two types of soil, and four water conditions, with three soybean seedlings per pot. Before the second pair of cotyledon leaves of the seedlings fully expanded (V2 stage), all treatments were maintained with adequate water. Drought treatment began at the V2 stage, with each treatment having three replicates. Soil moisture content was controlled and monitored using a soil moisture metre ECH2OTE/EC-TM (EM-50, Decagon, WA, USA) and the weighing method.

The drought treatments in the experiment consisted of withholding water for 0, 1, 3, and 5 days, corresponding to the control group (CK), mild drought (LD), intermediate drought (MD), and serious drought (SD), respectively. The soil moisture content in the CK, LD, MD, and SD treatments was 70%–80%, 50%–60%, 40%–50%, and 30%–40% of the field moisture content, respectively. The sampling time was at the end of the three-day treatment period, between 8:00 and 9:00 a.m. The samples were taken from the second-to-last pair of cotyledon leaves and the third-to-last pair of cotyledon leaves. After sampling, the samples were rapidly frozen in liquid nitrogen and then stored in a −80°C ultra-low temperature refrigerator.

### Assessment of physiological and biochemical indicators

The content of MDA, GSH, and Pro in leaves, as well as the activities of SOD, 6PGDH, sucrose synthase (synthesis direction) (SS-II), sucrose synthase (decomposition direction) (SS-I), NR, and GS were determined using a kit (Grace Biotechnology, Suzhou, China).

Weigh 0.1 g leaf tissue, add 1 ml corresponding index extract, and homogenize at 4°C or in an ice bath. Centrifuged at 12 000 rpm and 4°C for 10 min. The supernatant was placed on ice for testing.

The content of MDA in leaves was determined. Add 400 μl of extract and 600 μl of the reaction solution to the centrifuge tube, then place in a water bath at 95°C for 30 min, then cool, and centrifuge at 25°C at 12 000 g for 10 min. The absorbance was read at 532 nm and 600 nm, respectively.

The activity of SOD in leaves was determined. The crude enzyme solution and the specified reagent were added to the centrifuge tube, and then the absorbance was measured at 450 nm after standing for 30 min at 25°C in the dark.

The content of GSH in leaves was determined. The sample and the specified reagent (all reagents need to be kept in a water bath at 25°C for 10 min before use) were added in turn to a 1 ml glass cuvette and immediately mixed. After standing for 5 min, the absorbance values A’_GSH_ and A_GSH_ were read at the wavelength of 412 nm (A’_GSH_ was the test tube and A_GSH_ was the reference tube).

The activity of SS-II in leaves was determined. Add the sample and the specified reagent, and place in a 37°C water bath for 20 min. Then add the specified reagent after mixing, place in a 95°C water bath, and boil for 10 min. After cooling to room temperature, add the specified reagent again, mix, and place in a 95°C water bath for 20 min. Finally, after cooling, determine the absorbance at 480 nm.

The activity of SS-I of leaves was determined. 40 μl of the specified reagent was added to the determination tube, 40 μl of distilled water was added to the control tube, and then 10 μl of samples were added respectively, 37°C water bath for 30 min, then 95°C water bath for 5 min, and then the specified reagent was added respectively, 95°C water bath for 10 min. After taking out the solution to room temperature, 200 μl of distilled water was added, mixed, and the absorbance of each tube was measured at 540 nm.

The activities of NR and GS in leaves were determined. The NR activity was determined, and the crude enzyme solution and the specified reaction solution were added according to the kit instructions, and the water bath was shaded at 30°C for 30 min. Then the mixed reaction solution was added and reacted at 30°C for 15 min in the dark. The absorbance A’_NR_ and A_NR_ were measured at 530 nm (A’_NR_ was the measuring tube and A_NR_ was the reference tube). The GS activity was determined according to the kit instructions. The enzyme solution and three specified reaction solutions were added, and the water bath was 37°C for 30 min. The fourth reaction solution was added, mixed, reacted for 2 min, 8000 g, and centrifuged at 4°C for 10 min. The absorbance A’_GS_ and A_GS_ were measured at 540 nm (A’_GS_ is the measuring tube, A_GS_ is the reference tube).

The Pro content in the leaves was determined. 0.1 g frozen samples were weighed, 1 ml of the extract was added, homogenized in an ice bath, transferred to a 1.5 ml EP tube, and extracted in a water bath at 90°C for 10 min. The supernatant was drawn and cooled to be measured. A 150 μl sample was added to the determination tube, 150 μl of distilled water was added to the blank tube, and then the specified reagents were added, respectively. The samples were heated at 95°C water bath for 30 min, cooled to room temperature, and the absorbance values A’_Pro_ and A_Pro_ were read at 520 nm immediately (A’_Pro_ was the determination tube and A_Pro_ was the blank tube).

The activity of 6PGDH in leaves was determined. 0.1 g of the frozen sample was weighed, 1 ml of extract was added, homogenized in an ice bath, centrifuged at 4°C and 12 000 g for 15 min, and the supernatant was taken and placed on ice for testing. The specified reagent was mixed with the sample. The A_1_ value was read immediately at 450 nm at 25°C, and the A_2_ value was read after 20 min.

### Data and analysis

The data in the paper were created as histograms using Microsoft Office Excel 2019 and analysed using IBM SPSS Statistics (version 22.0: IBM Corporation, Armonk, USA) for Duncan’s single-factor variance analysis.

## Results

### The effects of drought stress on the MDA levels of soybean seedlings under different soil textures during the seedling stage

**Figure 1.**
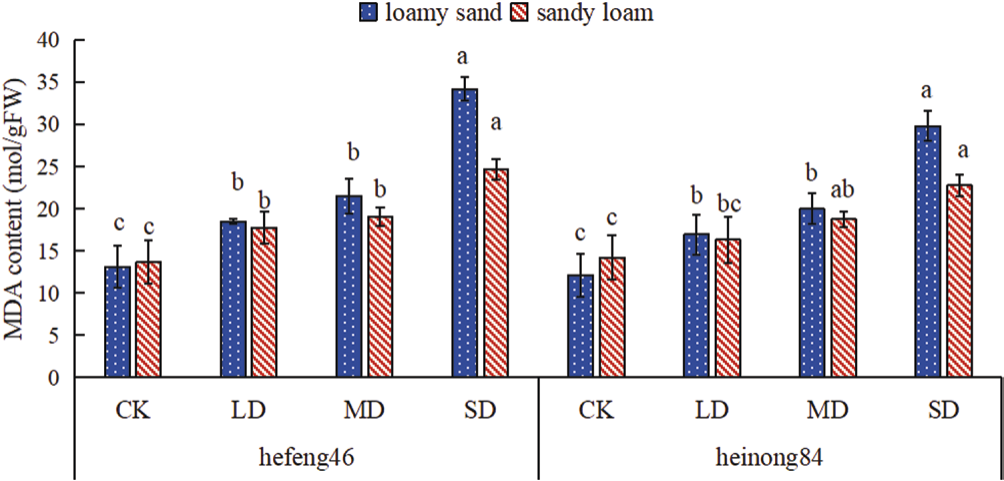
Effect of soil type on the MDA content of soybeans under drought stress. CK represents the control, LD represents mild drought, MD represents intermediate drought, and SD represents serious drought. Different letters indicate significant differences in the MDA content of soybeans under different water treatments in the same soil texture, with significance at *P* < .05.

In sandy loam and loamy sand, as the duration of drought increased, the MDA content in the leaves of both soybean varieties also increased. In CK, the MDA content of both soybean varieties in loamy sand was lower than that in sandy loam; however, the MDA content of both varieties was higher in loamy sand than in sandy loam when the duration of drought was prolonged. In loamy sand, compared to CK, the MDA content of the leaves of hefeng46 and heinong84 soybean varieties in the SD dramatically increased, with increases of 160% and 146%, respectively. In sandy loam, the MDA content of the leaves of hefeng46 in the SD prominently exceeded that of the CK, with an increase of 80%, the MDA content of the leaves of heinong84 did show a significant difference (Fig. 1, see Supplementary Table S1).

### Effect of drought stress on the SOD activity of soybean seedling leaves under different soil substrates

**Figure 2.**
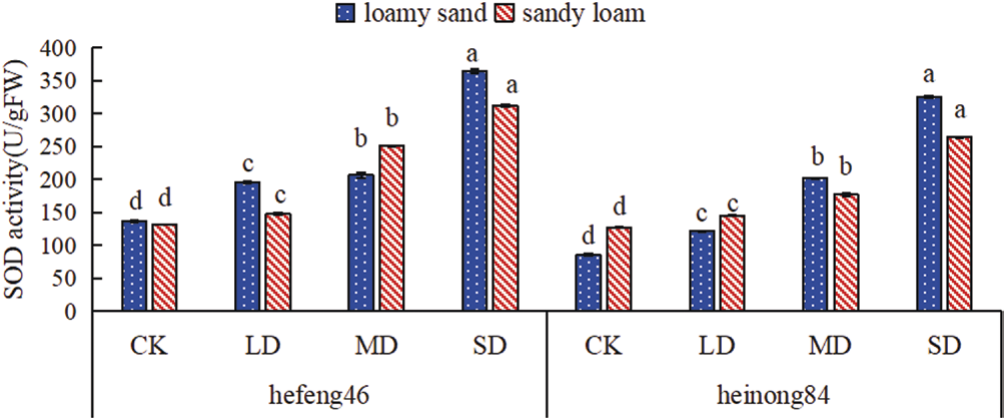
Effect of soil texture on soybean SOD activity under drought stress. CK represents the control, LD represents mild drought, MD represents intermediate drought, and SD represents serious drought. Different letters indicate significant differences in the SOD activity of soybeans under different water treatments in the same soil texture, with significance at *P* < .05.

In both soil matrices, as the prolongation of drought treatment increased, the SOD activity of hefeng46 and heinong84 soybeans also increased. In hefeng46 soybeans, the SOD activity in treatments in loamy sand (excluding the MD) was higher than in sandy loam; in heinong84 soybeans, the SOD activity in sandy loam in the CK and LD treatments was higher than in loamy sand, while the SOD activity in loamy sand in the MD and SD was higher than in sandy loam. It can also be known from Fig. 2 that in loamy sand soil, the SOD activity of hefeng46 and heinong84 soybeans treated with SD compared to CK significantly increased, with increases of 167% and 282%, respectively; in sandy loam soil, the SOD activity of hefeng46 and heinong84 soybeans treated with SD compared to CK significantly increased, with increases of 138% and 108%, respectively (Fig. 2, see Supplementary Table S1).

### The effect of drought stress on the GSH content in soybean seedling leaves under different soil matrices

**Figure 3.**
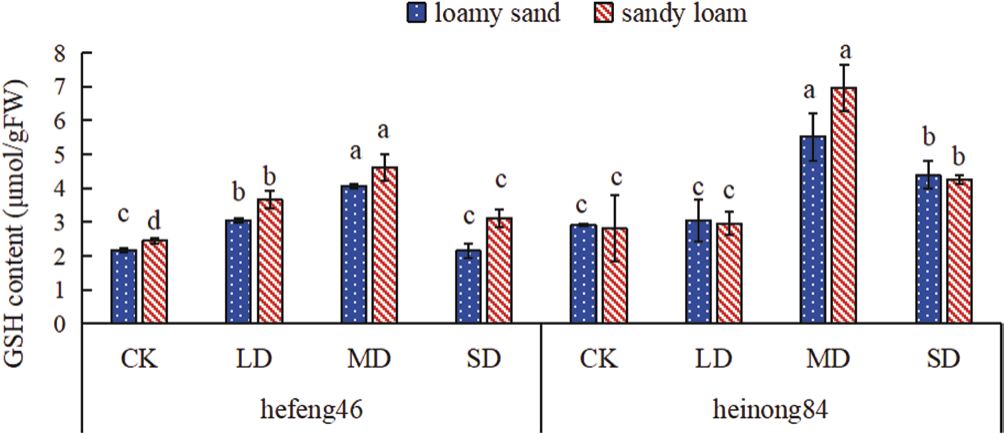
The effect of soil type on GSH content in soybean seedlings under four moisture treatments. CK represents the control, LD represents mild drought, MD represents intermediate drought, and SD represents serious drought. Different letters suggest significant differences in the GSH content of soybeans in the same type of soil under four moisture treatments, with significance set at *P* < .05.

In both soils, as the duration of drought increased, the GSH content of both soybean varieties increased to a peak at MD and then decreased. In the four moisture treatments of hefeng46 soybeans, the GSH content in sandy loam soil is higher than in loamy sand soil, whereas in the heinong84 soybeans, the GSH content in loamy sand soil is generally lower than in sandy loam soil (except for treatment MD). In loamy sand, the GSH content in hefeng46 soybeans at MD was significantly increased compared to CK, by 87%, while there was significance in GSH content in heinong84 soybeans, with an increase of 90% at MD compared to CK; in sandy loam, the GSH content in both hefeng46 and heinong84 soybeans at MD was significantly increased compared to CK, by 47% and 148%, respectively (Fig. 3, see Supplementary Table S1).

### The effect of drought stress on the Pro content in soybean seedling leaves under different soil matrices

**Figure 4.**
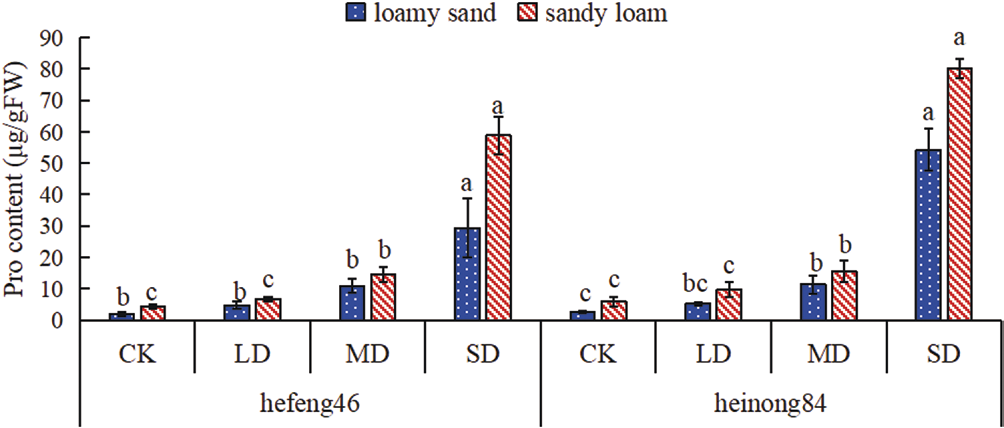
The effect of four moisture conditions on the Pro content in soybean seedlings under different soil matrices. CK represents the control, LD represents mild drought, MD represents intermediate drought, and SD represents serious drought. Different letters suggest striking differences in the Pro content of soybeans under the four moisture treatments of the same soil type, with significance set at *P* < .05.

In both soil types, as the duration of drought increased, the content of Pro also increased in both soybean varieties, and the content of Pro in soybeans under the four moisture treatments in sandy loam soil was higher. In loamy sand, the Pro content in both hefeng46 and heinong84 soybeans significantly increased under SD compared to CK, with increases of 1431% and 1924%, respectively. In sandy loam, the Pro content in both hefeng46 and heinong84 soybeans significantly increased under SD compared to CK, with increases of 1275% and 1252%, respectively (Fig. 4, see Supplementary Table S1).

### The effect of drought stress on the 6PGDH activity in soybean seedling leaves under different soil matrices

**Figure 5.**
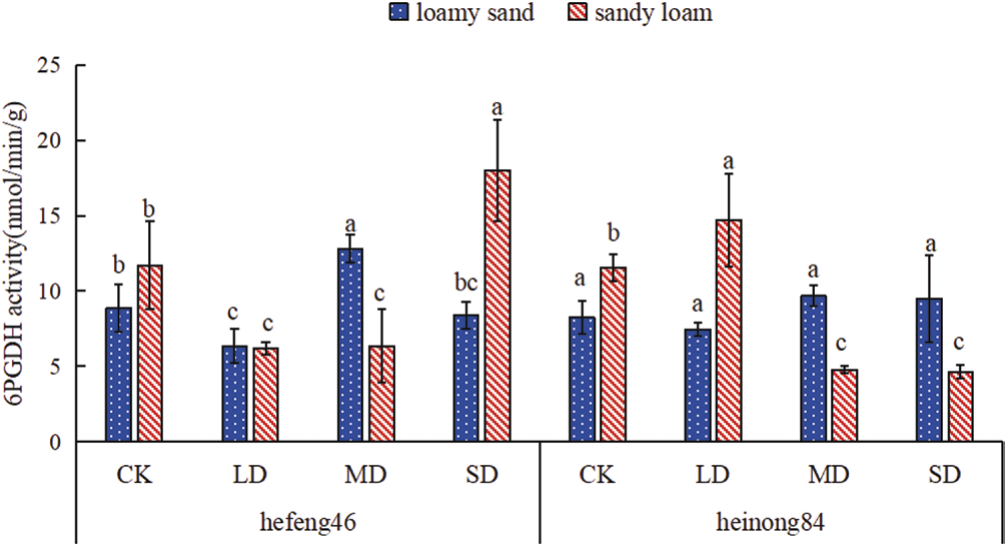
The effect of different moisture treatments on the 6PGDH activity in soybean seedlings under different soil matrices. CK represents the control, LD represents mild drought, MD represents intermediate drought, and SD represents serious drought. The different letters stand for the remarkable differences (*P* < .05) in the 6PGDH activity of soybeans under different moisture treatments of the same soil type.

In loamy sand, the 6PGDH activity in both hefeng46 and heinong84 soybeans displayed a trend of first dropping and then rising, and then descending with the duration of drought treatment. The peak activity was observed in MD. Compared to CK, the 6PGDH activity of hefeng46 MD increased sensibly by 44%, and there was an unobvious difference in the 6PGDH activity of heinong84 soybeans under various drought treatments. In sandy loam, the 6PGDH activity in hefeng46 soybeans displayed a trend of first downswings and then adding with the duration of drought treatment, with the peak activity observed in SD. Compared to the MD, the 6PGDH activity in the SD treatment was dramatically enhanced by 1.9 times. The 6PGDH activity in heinong84 soybeans expressed a trend of first enhancing and then descending with the duration of drought treatment, with the peak activity observed in the LD treatment. Compared to the LD treatment, the 6PGDH activity in the SD prominently descended by 69% (Fig. 5, see Supplementary Table S1).

### The effect of four moisture conditions on the activity of sucrose synthase in soybean seedlings under different soil matrices

**Figure 6.**
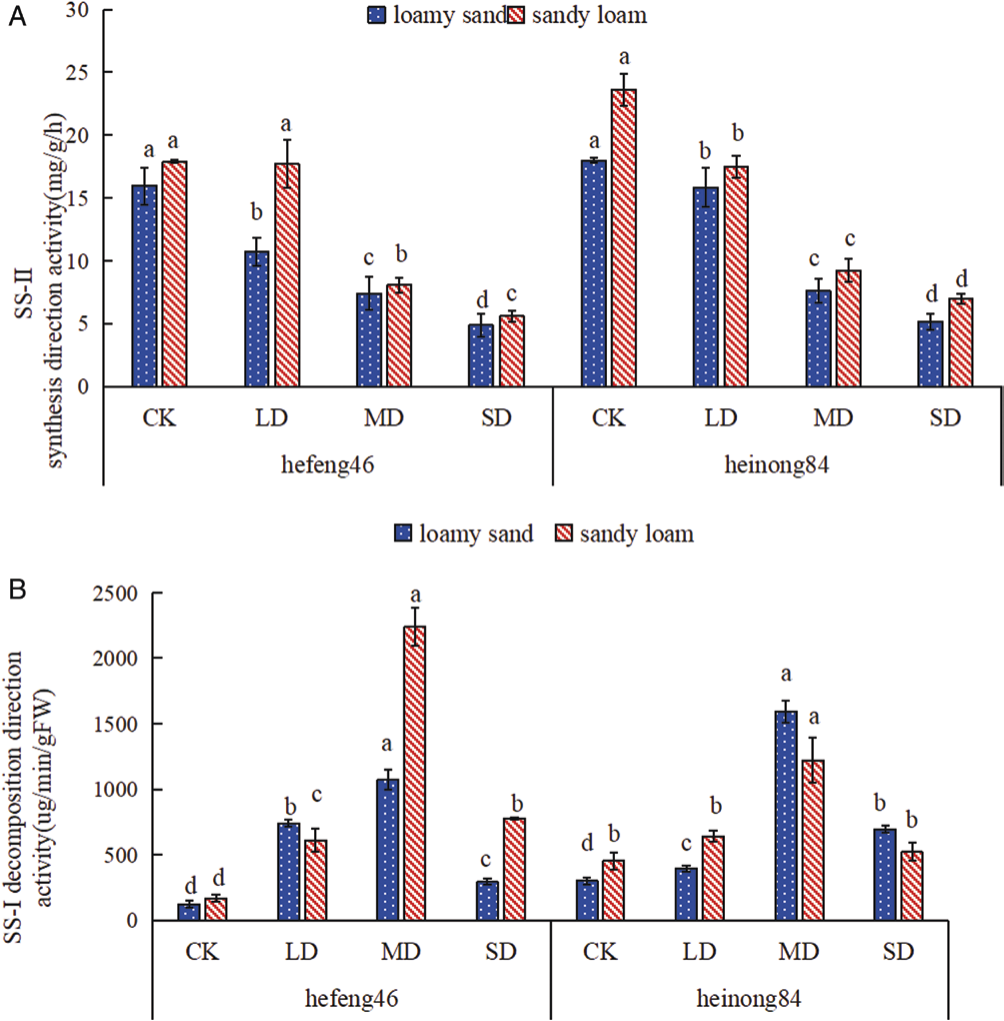
(A) The effect of soil types on the synthesis-oriented activity of soybean seedling SS-II under drought stress. (B) The effect of soil types on the decomposition-oriented activity of soybean seedling SS-I under four moisture conditions. CK represents the control, LD represents mild drought, MD represents intermediate drought, and SD represents serious drought. Different characters declare remarkable differences (*P* < .05) in the activity of soybean SS-II and SS-I in the same type of soil under four moisture conditions.

In both soil types, as the duration of drought expanded, the SS-II activity of both soybean varieties showed a decreasing trend, but the SS-II activity in sandy loam was more active. In loamy sand, the activity of SS-II synthase in hefeng46 soybean was prominently downgraded in SD compared to the CK, by 70%, while in heinong84 soybean, the activity of SS-II synthase was significantly downgraded in SD compared to the CK, by 61%; in sandy loam, the activity of SS-II synthase in hefeng46 soybean was significantly reduced by 69% in SD compared to the CK, and in heinong84 soybean, the activity of SS-II synthase was prominently downgraded by 70% in the SD compared to the CK (Fig. 6A, see Supplementary Table S1).

In both soil types, the activity of soybean SS-I synthase showed an increasing trend followed by a decline with the increase in duration of drought stress, with a peak observed in MD. In hefeng46 soybeans, the SS-I activity in sandy loam soil is more active (except for LD), while in heinong84 soybeans, the SS-I activity in sandy loam soil is more active in the CK and LD, but the opposite is true in MD and SD. In loamy sand, the activity of SS-I synthase in hefeng46 soybean was dramatically raised in the MD compared to the CK, by 778%, while in heinong84 soybean, the activity of SS-I synthase was significantly enhanced in MD compared to the CK, by 430%; in sandy loam, the activity of SS-I synthase in hefeng46 soybean was sensibly enhanced in the MD compared to the CK, by 1247%, and in heinong84 soybean, the activity of SS-I synthase was prominently increased in the MD compared to the CK, by 169% (Fig. 6B, see Supplementary Table S1).

### The effect of drought stress on the activity of soybean leaf nitrate reductase under different soil matrices

**Figure 7.**
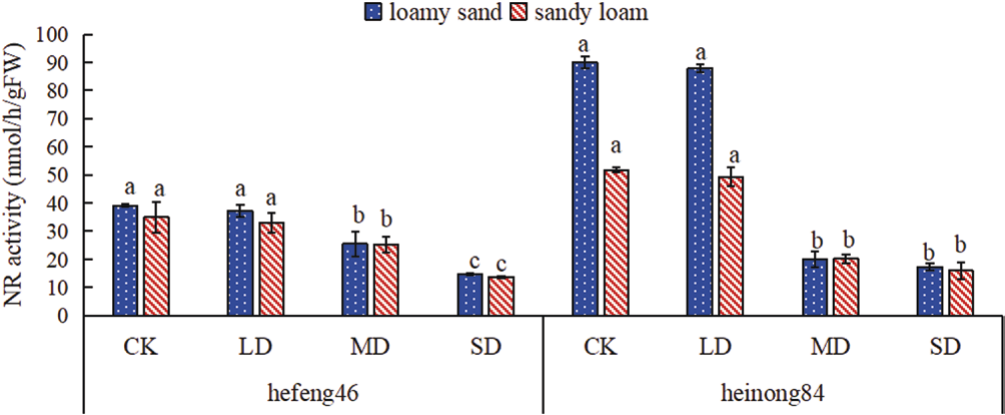
The effect of soil types on the activity of soybean seedling NR under four moisture conditions. CK represents the control, LD represents mild drought, MD represents intermediate drought, and SD represents serious drought. Different characters declare remarkable differences (*P* < .05) in the activity of soybean NR in the same type of soil under four moisture conditions.

In both soil types, as moisture decreases, the NR activity of hefeng46 and heinong84 soybeans lowers, and the NR activity in all four moisture treatments is higher in loamy sand. In loamy sand, the activity of NR in hefeng46 soybean was prominently lowered in the SD compared to the CK, by 62%, while in heinong84 soybean, the activity of NR was prominently reduced in the SD compared to the CK, by 81%; in sandy loam, the activity of NR in hefeng46 soybean was dramatically reduced in the SD compared to the CK, by 61%, and in heinong84 soybean, the activity of NR was dramatically lowered in the SD compared to the CK, by 69% (Fig. 7, see Supplementary Table S1).

### The effect of drought stress on the activity of soybean leaf GS under different soil matrices

**Figure 8.**
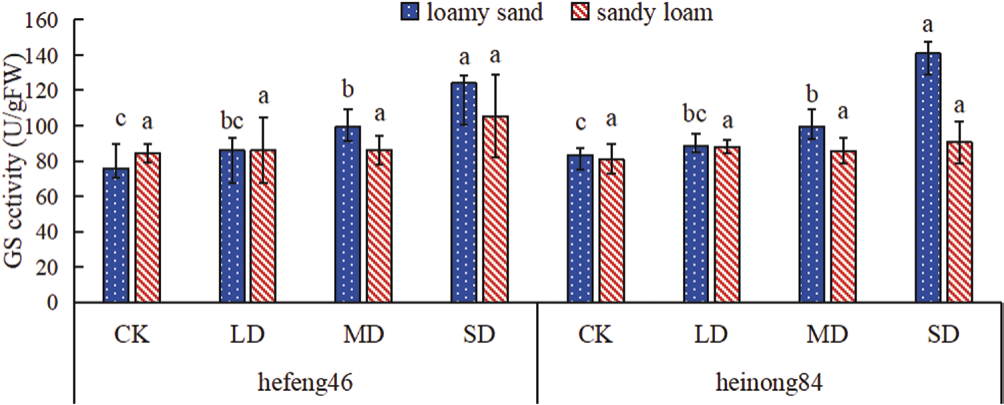
The effect of soil types on the activity of soybean seedling GS under drought stress. CK represents the control, LD represents mild drought, MD represents intermediate drought, and SD represents serious drought. Different characters declare remarkable differences in the activity of soybean GS in the same type of soil under different degrees of drought stress, with significance at *P* < .05.

In loamy sand, the activity of GS in hefeng46 and heinong84 soybeans increased with the duration of water stress; in sandy loam, there was no remarkable difference in the activity of GS between hefeng46 and heinong84 soybeans; under different moisture conditions, the GS activity is more active in loamy sand for both soybean varieties. In loamy sand, the activity of GS in hefeng46 and heinong84 soybeans was dramatically raised in the SD compared to the CK, by 64% and 69%, respectively; in sandy loam, there was no remarkable difference in the activity of GS between the various treatments of the two soybean varieties (Fig. 8, see Supplementary Table S1).

## Discussion

There is quite a few research that shows that the MDA content in plant tissues will increase when plants are subjected to drought (Zhou *et al*. 2022b; Shehzad *et al*. 2023). In this experiment, as the duration of drought increased, the MDA content of both soybean varieties also increased, consistent with previous research results. Studies have shown that sandy loam soil has higher water-holding capacity and higher nutrient efficiency than loamy sand soil (Malaka *et al*. 2022). As can be seen from Fig. 1, under sufficient water conditions, the MDA content of both soybean varieties in sandy loam was higher than that in loamy sand; however, when both varieties were subjected to drought stress, the MDA content of soybeans grown in loamy sand was higher, consistent with the results of Zhou’s study on the effects of drought stress on florescence of soybean under different soil textures (Zhou *et al*. 2022b).

When plants are subjected to environmental stress, they produce high concentrations of ROS that damage cell structures. When plants are exposed to drought stress, SOD activity changes to act as the first line of defense against ROS to alleviate damage (Zhou *et al*. 2022a). A large amount of studies indicates that the activity of SOD in plants markedly increases with the level of drought stress (He *et al*. 2020; Ru *et al*. 2023). When facing non-biological stress, glutathione in the plant defense system is considered to be one of the most important metabolites. Under normal stress levels, plants induce the synthesis of GSH to stabilize the ratio between oxidized glutathione and reduced glutathione. But then under utmost stress conditions, the synthesis of GSH may be repressed (Koh *et al*. 2021). Currently, there have been numerous studies that have concluded that drought promotes the synthesis of Pro in plants while inhibiting its degradation. By regulating the osmotic pressure, Pro helps to stabilize the antioxidant system and protect the integrity of cell membranes, thereby mitigating the damage caused by ROS to plants (Ferreira Júnior *et al*. 2018; Zhou *et al*. 2023). In this experiment, as the level of drought stress increased, the activity of SOD and the content of Pro in the leaves of the two soybean varieties increased, while the content of GSH increased under mild and intermediate drought stress and decreased under SD stress, consistent with the results of previous research. Under water stress, compare to sandy loam, the changes in the activity of SOD and the content of Pro and GSH in hefeng46 and heinong84 are more prominently in loamy sand, while the content of GSH in heinong84 change dramatically (Figs. 2–4). These results prove that under water stress, the changes in the activity of antioxidant enzymes and the content of non-enzymatic antioxidants in varieties hefeng46 and heinong84 in sandy loam are smaller, indicating that the growth of soybeans is affected to a lesser extent. The reason why the content of GSH in the variety hefeng46 under four water treatments was higher in sandy loam may be due to differences in the variety.

The PPP is an important route of plant sugar metabolism. 6PGDH is one of the key enzymes in the PPP and also one of its rate-limiting enzymes, playing a role in catalysing the dehydrogenation of 6-phosphogluconate to produce 5-phospho-d-ribulose. Research has found that under conditions of 10% water loss, the activity of 6PGDH in the fruiting bodies of black fungus is lower than under normal water conditions, but as the degree of water loss increases, the activity of 6PGDH in the fruiting bodies of black fungus significantly increases (Ma *et al*. 2015). As can be seen from Fig. 5, the activities of 6PGDH both hefeng46 and heinong84 in loamy sand and hefeng46 in sandy loam under mild drought conditions are lower than under normal water conditions. Moreover, the activity of 6PGDH in hefeng46 in sandy loam increases with the duration of drought, which is consistent with the results of previous research. Studies have shown that sandy loam soil has higher water-holding capacity and higher nutrient efficiency than loamy sand soil (Malaka *et al*. 2022). Therefore, the activity of 6PGDH in the mild drought of heinong84 in sandy loam is higher than under normal water conditions. The reason for this phenomenon may be the difference in soybean varieties and the stronger water retention capacity of sandy loam. As the duration of drought increases, the activity of 6PGDH both hefeng46 in loamy sand and heinong84 in sandy loam enhances first and then decreases. The reason for this result may be that intermediate and serious droughts lead to significant water deficits, causing the gradual inactivation of 6PGDH. Sucrose synthase catalyses the reversible reaction: uridine diphosphate + sucrose ↔ uridine diphosphate glucose + fructose. Studying the activity of SS-II and SS-I is of vital significance for the synthesis and decomposition of plant sucrose. Explorations have expressed that drought stress can improve the ability of sucrose to fructose conversion, thereby upgrading the osmotic regulating ability under drought stress (Du *et al*. 2020b). However, the results of this experiment displayed that drought stress significantly diminishes the activity of SS-II, with the impact on the activity of SS-I showing an initial significant increase, followed by a peak and then suppression. The phenomenon may occur because light and intermediate drought stress promote the decomposition of sucrose and inhibit the synthesis of sucrose, while severe drought stress eventuates severe water shortage in plants, affecting the activity of SS-I (Fig. 6A and B). Many explorations have suggested that drought stress adds the content of nitric oxide in plant tissues, and the activity of NR, which catalyses the conversion of nitrate to nitrite, may be inhibited (Lau *et al*. 2021). Studies have shown that when cotton is encountered to water stress, the activity of NR decreases. This discovery is consistent with the outcomes of the research, which showed that the activity of NR in hefeng46 and heinong84 soybeans decreased rapidly in loamy sand under drought stress, while the decrease trend was slower in sandy loam (Zahoor *et al*. 2017). The reason for the slower decrease in NR activity in sandy loam compared to loamy sand may be that sandy loam has a higher efficiency of nutrient utilization and better water retention capacity, and under drought conditions, the impact on soybean growth is smaller (Fig. 7). Nitrate can be catalysed by NR to form nitrite, which is then degraded into ammonium by the enzyme nitrite reductase. GS can convert glutamate and NH_4_^+^ into glutamine. Studies have shown that under drought stress, the activity of GS in plant leaves decreases, but the application of nitrogen and regulators significantly improves the activity of GS in soybeans, which helps to maintain the relatively stable nitrogen assimilation ability of plants under drought conditions. The enhancement of GS activity under drought stress is beneficial for improving the drought resistance of plants (Yu *et al*. 2020; Sahay *et al*. 2021; Gai *et al*. 2023). The results of the experiment showed that in loamy sand, drought stress increased the activity of GS in both hefeng46 and heinong84 soybean varieties, while in sandy loam, there was no remarkable difference in the activity of GS between the two soybean varieties, indicating that soybeans are suitable for planting in sandy loam under drought stress (Fig. 8).

The above results indicate that under water stress, compared to sandy loam, the osmotic regulation in hefeng46 and heinong84 in loamy sand is higher, indicating that when soybeans in loamy sand are subjected to drought stress, their physiological and biochemical processes are more affected.

The limitation of this study is the small number of drought treatments and the large span of soil water content between treatments, which cannot better reflect the physiological metabolic changes of the two soybeans under drought stress.

## Conclusion

This study revealed the effects of two soil types on soybean physiology under drought stress, which is helpful to improve soybean drought resistance and optimize soil management. In both soil matrices, different levels of drought stress have affected the physiological and biochemical processes of soybean seedlings. Based on the comprehensive analysis of membrane lipid peroxidation levels, activities of antioxidant enzymes, concentrations of non-enzymatic antioxidants in soybean leaves, and changes in key enzymes of carbon and nitrogen metabolism, it can be seen that under drought stress, hefeng46 and heinong84 grow more adaptively in matrix sandy loam.

Conflict of interest: All authors declared that there is no conflict of interest.

## Supplementary Material

plaf026_suppl_Supplementary_Tables_S1

## Data Availability

The data underlying this article are available in the article and in its online supplementary material.
